# Early intensification of backyard poultry systems in the tropics: a case study

**DOI:** 10.1017/S175173112000110X

**Published:** 2020-11

**Authors:** C. Chaiban, T. P. Robinson, E. M. Fèvre, J. Ogola, J. Akoko, M. Gilbert, S. O. Vanwambeke

**Affiliations:** 1 Georges Lemaître Centre for Earth and Climate Research, Earth and Life Institute, Université catholique de Louvain, UCLouvain, 1348 Louvain-la-Neuve, Belgium; 2 Spatial Epidemiology Lab (SpELL), Université Libre de Bruxelles, 1050 Brussels, Belgium; 3 Livestock Information, Sector Analysis and Policy Branch (AGAL), Food and Agriculture Organization of the United Nations (FAO), Viale delle Terme di Caracalla, 00153 Rome, Italy; 4 International Livestock Research Institute (ILRI), 00100 Nairobi, Kenya; 5 Institute of Infection and Global Health (IGH), University of Liverpool, Liverpool L7 3EA, UK; 6 County Directorate of Veterinary Services, Bungoma County 50200, Kenya; 7 Fonds National de la Recherche Scientifique (FNRS), 1000 Brussels, Belgium

**Keywords:** livestock intensification, farm typology, small-scale production, poultry production, Kenya

## Abstract

Poultry production is an important way of enhancing the livelihoods of rural populations, especially in low- and middle-income countries (**LMICs**). As poultry production in LMICs remains dominated by backyard systems with low inputs and low outputs, considerable yield gaps exist. Intensification can increase poultry productivity, production and income. This process is relatively recent in LMICs compared to high-income countries. The management practices and the constraints faced by smallholders trying to scale-up their production, in the early stages of intensification, are poorly understood and described. We thus investigated the features of the small-scale commercial chicken sector in a rural area distant from major production centres. We surveyed 111 commercial chicken farms in Kenya in 2016. We targeted farms that sell the majority of their production, owning at least 50 chickens, partly or wholly confined and provided with feeds. We developed a typology of semi-intensive farms. Farms were found mainly to raise dual-purpose chickens of local and improved breeds, in association with crops and were not specialized in any single product or market. We identified four types of semi-intensive farms that were characterized based on two groups of variables related to intensification and accessibility: (i) remote, small-scale old farms, with small flocks, growing a lot of their own feed; (ii) medium-scale, old farms with a larger flock and well located in relation to markets and (iii) large-scale recently established farms, with large flocks, (iii-a) well located and buying chicks from third-party providers and (iii-b) remotely located and hatching their own chicks. The semi-intensive farms we surveyed were highly heterogeneous in terms of size, age, accessibility, management, opportunities and challenges. Farm location affects market access and influences the opportunities available to farmers, resulting in further diversity in farm profiles. The future of these semi-intensive farms could be compromised by several factors, including the competition with large-scale intensive farmers and with importations. Our study suggests that intensification trajectories in rural areas of LMICs are potentially complex, diverse and non-linear. A better understanding of intensification trajectories should, however, be based on longitudinal data. This could, in turn, help designing interventions to support small-scale farmers.

## Implications

Agricultural intensification occurs everywhere but intensification in smallholder livestock production in low- and middle-income countries is poorly understood. We characterized the semi-intensive production systems of chicken in a remote rural area of Kenya. Our findings highlight the heterogeneity of farm types in the small-scale commercial sector and the range of constraints faced by the farmers. Interventions aimed at poultry production development should consider the great diversity in profiles and constraints.

## Introduction

Poultry constitutes a significant source of protein and income in low- and middle-income countries (**LMICs**) (Guèye, 1998; Whyte, 2002; FAO, 2018). Demand per capita for poultry products is predicted to increase by 100% from 2000 to 2030 in those countries (Robinson and Pozzi, 2011). Population growth and changing consumption patterns linked to urbanization and increasing wealth drive this growth in demand. The increase in demand for poultry products, in turn, drives structural changes in the sector. These may take the form of expansion, with more people producing, intensification of production and increased trade in products.

In LMICs, two main types of poultry production coexist. Intensive systems are large-scale farms raising specialized breeds of broilers and layers (Sonaiya and Swan, 2004; FAO, 2008; Thieme *et al.*, 2014). They raise genetically similar animals for commercial purposes, buy day-old chicks and use commercial feed (FAO, 2018). Backyard, dual-purpose systems, conversely, keep a small number of indigenous chickens, generally less than 50 birds. These chickens are typically raised in low-input, low-output, free-ranging systems where production is dedicated mainly to home consumption but also to provide the possibility to raise cash in case of emergency (Guèye, 1998; Whyte, 2002; Thieme *et al.*, 2014; Mwobobia *et al.*, 2016; Alders *et al.*, 2018). Backyard chickens mainly scavenge but feed supplements may be provided (Sonaiya and Swan, 2004; Thieme *et al.*, 2014). Vaccination is rarely implemented and replacement stock comes generally from natural incubation (Thieme *et al.*, 2014). Backyard chickens are exposed to predators, diseases, theft and management difficulties (Mack *et al.*, 2005). Currently, smallholders raising poultry in backyard systems make up the large majority of producers in low-income countries (Gilbert *et al.*, 2015). They generally have a low productivity (Okeno *et al.*, 2012), and both their productivity and income could be improved by intensification (Magothe *et al.*, 2010; Ochieng *et al.*, 2011; Okeno *et al.*, 2012), that is, ‘the increased use of external inputs and services to increase the output quantity and/or value per unit input’ (Bebe *et al.*, 2002).

While increasing demand for poultry products drives intensification at the country level, this may not benefit all producers equally. In LMICs, the poorest and smallest producers are frequently found to benefit less from the overall economic growth and the transformation of the market structure than more resources endowed producers (FAO, 2018). Improvements in productivity may be limited when the innovations are not within the physical and economic resources of smallholders (Aklilu *et al.*, 2007) who then struggle to compete with large-scale intensive systems. While extensive smallholders as well as large-scale intensive systems have been well described (Sonaiya and Swan, 2004; FAO, 2008 and 2014; Alders and Pym, 2009; Alders *et al.*, 2018), little is known about what characterizes the initial steps towards intensification; the early adoption of improved means of production, leading to increases in productivity and profitability. Presumably, this gives rise to semi-intensive systems (50 to 200 birds), which have been partially described as using combinations of commercial breeds, crossbred chickens (*i.e.* crosses between indigenous and exotic breeds (Rege *et al.*, 2011)) and indigenous chickens (Kingori *et al.*, 2010; Thieme *et al.*, 2014; Alders *et al.*, 2018). The birds may scavenge during the day but are confined overnight. Supplementation may include commercial feed and veterinary services are usually provided (Thieme *et al.*, 2014). Semi-intensive farmers may raise chickens alongside other livestock (Guèye, 2002; Thieme *et al.*, 2014). Their management practices and the constraints they face are heterogeneous (Thieme *et al.*, 2014). Gaining an understanding of the diversity among semi-intensive poultry producers – the different practices adopted, opportunities taken and constraints faced – and understanding different production strategies, is important if we are to realize farmers’ potential for growth. This could, in turn, help promote conditions conducive to economically sustainable intensification and to maximize the contribution of the poultry sector to improving livelihoods.

Kenya is a typical LMIC, in which consumption of poultry meat is predicted to increase from 54.8 in 2000 to 164.6 metric tonnes per year in 2030 (Robinson and Pozzi, 2011). Poultry production is still largely extensive in western Kenya but semi-intensive production is also encountered. The main features of the latter, however, are poorly known.

This study aimed to better understand the characteristics of systems of intermediate intensification as they exist in LMIC rural areas. We surveyed small-scale commercial chicken farms in an area distant from major Kenyan urban centres to characterize the diversity of emerging small-scale commercial chicken farms and developed a typology based on the inputs, outputs and spatial constraints.

## Material and methods

### Field survey

A field survey was conducted from April to July 2016 in an area of approximately 880 km^2^ in Busia, Bungoma and Kakamega counties (Supplementary Material Figure S1), to collect data about farm characteristics. This area borders Uganda and is distant from the major urban centres (e.g. Nairobi, Kisumu), though the recent devolution of Kenya created an urbanization impetus in many small towns which is generating market opportunities (Evans, 1992). We conducted a census of all commercial chicken farms following a snowball sampling approach: spiralling out of one first sub-location (smallest administrative unit in Kenya) and going from one assistant chief to another to reach farmers (Supplementary Material Figure S1).

We selected commercial chicken farms defined as those which sell most of their production, owning a minimum of 50 chickens, at least partly confined and provided with feed (Thieme *et al.*, 2014). Backyard flocks in which commercial activity was secondary were excluded. An administrative officer established a list of candidate farms and we confirmed that each one met the inclusion criteria upon visiting them. Each farm was visited with the help of either the assistant chief (sub-location administrative officer) or a village elder (Zone A in Supplementary Material Figure S1 – first sampling method). In five sub-locations from Nambale wards (Zone B1 in Supplementary Material Figure S1), in 15 sub-locations from Amukura ward (Zone B2 in Supplementary Material Figure S1) and all sub-locations from Chakol ward (Zone B3 in Supplementary Material Figure S1), ward livestock production officers were consulted (Zones B in Supplementary Material Figure S1 – second sampling method). This approach yielded a contiguous sampling area, scalable to the time required to proceed.

We interviewed 111 farms from 71 sub-locations. The analysis presented here focused on dual-purpose chicken production. We thus included 109 farms that kept dual-purpose chickens of local or improved breeds, of which two also kept broilers, four also kept layers and one kept layers and broilers. Two farms raised only layers and were excluded from the analysis.

To characterize commercial poultry farming, a questionnaire was administered requesting information on (i) general characteristics of the farm (time since commercial activity establishment, land ownership, land size, other income sources); (ii) characteristics of poultry production systems, farm stock and production (poultry breed and type, instant stock (including chicks, hens and cocks), birds slaughtered per year and typical slaughter weight (to obtain productivity), and input and output type: type of provider and output destination and prices); (iii) constraints faced by the farmers; and (iv) advantages and disadvantages of the farm location to study the spatial constraints of the farms. Farms were georeferenced. The questionnaire was adapted to poultry type: broiler, layer and dual-purposes breeds (local and improved breeds), and a separate questionnaire was administered for each type present on the farm.

### Statistical analysis

We used eight variables to define farm profiles (of farms raising dual-purpose breeds only), covering flock size, production, accessibility, breeds and chick source (Table 1A). To reduce correlation between variables and explore the data pattern, we performed a principal component analysis (**PCA**). We selected the first three principal components (**PCs**) according to the scree plot of eigenvalues and the total variability explained and used them in a clustering procedure. We tested a hierarchical and a non-hierarchical clustering method: Ward’s minimum variance clustering and K-means partitioning. Further details on the clustering methods are in the Supplementary Material.

**Table 1:** List of variables used in the principal component analysis and to define chicken farm profiles

Definition		Unit
(A) Variables used in the principal component analysis		
Instant stock	Log10 of the number of chickens at the time of interview (flock size)	–
Annual number of birds slaughtered	Log10 of the number of chickens sold per year (estimated by farmers or extrapolated)	–
Road accessibility	Travel time to the closest intersection between main roads (international and national paved trunk roads) and the rest of the road network (see Supplementary Material Table S1 and Figure 1)	min
Market accessibility	Travel time to the closest live bird market among the most cited by farmers (see Supplementary Material ‘Accessibility variables’ and Figure 1)	min
Local breeds	Farmer raising indigenous chickens or not	–
Improved breeds	Farmer raising improved breeds or not	–
Chick home produced	Farmer home-producing chicks or not	–
Chick bought	Farmer purchasing chicks or not	–
(B) Other variables used to define farm profiles		
Farm age	Time since commercial activity started	years
Farm size	Land use size (compound and cropland)	ha
Meat production	Annual meat production = annual number of birds slaughtered × live weight × dressing percentage (see Supplementary Material ‘Meat production variable’)	kg of meat/year
Meat productivity	Annual meat production by the instant stock	kg/bird/year
Egg production	Annual mean of the number of eggs sold in high and low season and at interview time	Number of eggs/year
Egg productivity	Annual egg production by the number of hens	Number of eggs/hen place/year
Live weights	Usual slaughtered weight of hen or cock estimated by farmers	kg
Feed types	Open question classified into three types: home-produced feed (referring to their own crops), raw products (referring to ingredients bought separately and mixed by farmers) or commercial feed	–
Products sold	Chickens, eggs and chicks	–
Prices of outputs	Classified by product (chicken, egg), sale type (wholesale, retail) and price type (usual, maximum, minimum price)	Kenyan shillings
Types of output destination	Open question classified into six destinations: market, trader, farm gate sales (neighbours, local schools or functions), farmers, restaurant (locally called ‘hotel’) or other (school, shop for eggs, slaughterhouse, butcher)	–
Advantages of farm location	Open question classified into five advantages: access to market, available market, access to feed, access to road, own crops used as feed	–
Disadvantage of farm location	Open question classified into six disadvantages: insufficient demand, low market access, low road access, low prices of output, disease and theft of chickens	–
Constraints	Open question classified into 11 constraints: feed cost, disease, lack of knowledge on chick management, lack of knowledge on poultry keeping management, lack of electricity, vaccine and drug cost, theft of chickens, predation, low prices of Ugandan eggs, lack and instability of market and lack of fund	–

We characterized the clusters into farm profiles with farm age and size, meat production and productivity, egg production and productivity, feed types, types of product sold and where, prices, advantages and disadvantages of farm location and constraints (Table 1B). We measured farm accessibility to the main roads and to markets in travel time as detailed in Supplementary Material (‘Accessibility variables’ and Table S1).

## Results

### Farm typology

The first three PCs had eigenvalues over one and represented 72% of the variability (Supplementary Material Figure S2a). The first PC (39%) correlated most with breed type, chick source, instant stock and annual number of birds slaughtered (Supplementary Material Figure S3b and f). The second PC (20%) distinguished farms by travel times to main markets and main roads, which were strongly correlated (Supplementary Material Figure S3b and d). The third axis (13%) slightly overlapped PC1 but was mostly associated with the source of chicks and flock size (represented by the instant stock) (Supplementary Material Figure S3d and f).

A balance of the within-cluster similarity and the number of clusters by both Ward and K-means clustering methods was best achieved by selecting four clusters (Supplementary Material Figure S2c and d). With the exception of seven farms, both clustering methods were concordant (Supplementary Material Figure S2b). The K-means results were preferred as farms spread more widely along the PCA dimensions (Supplementary Material Figure S2b).

**Figure 1:**
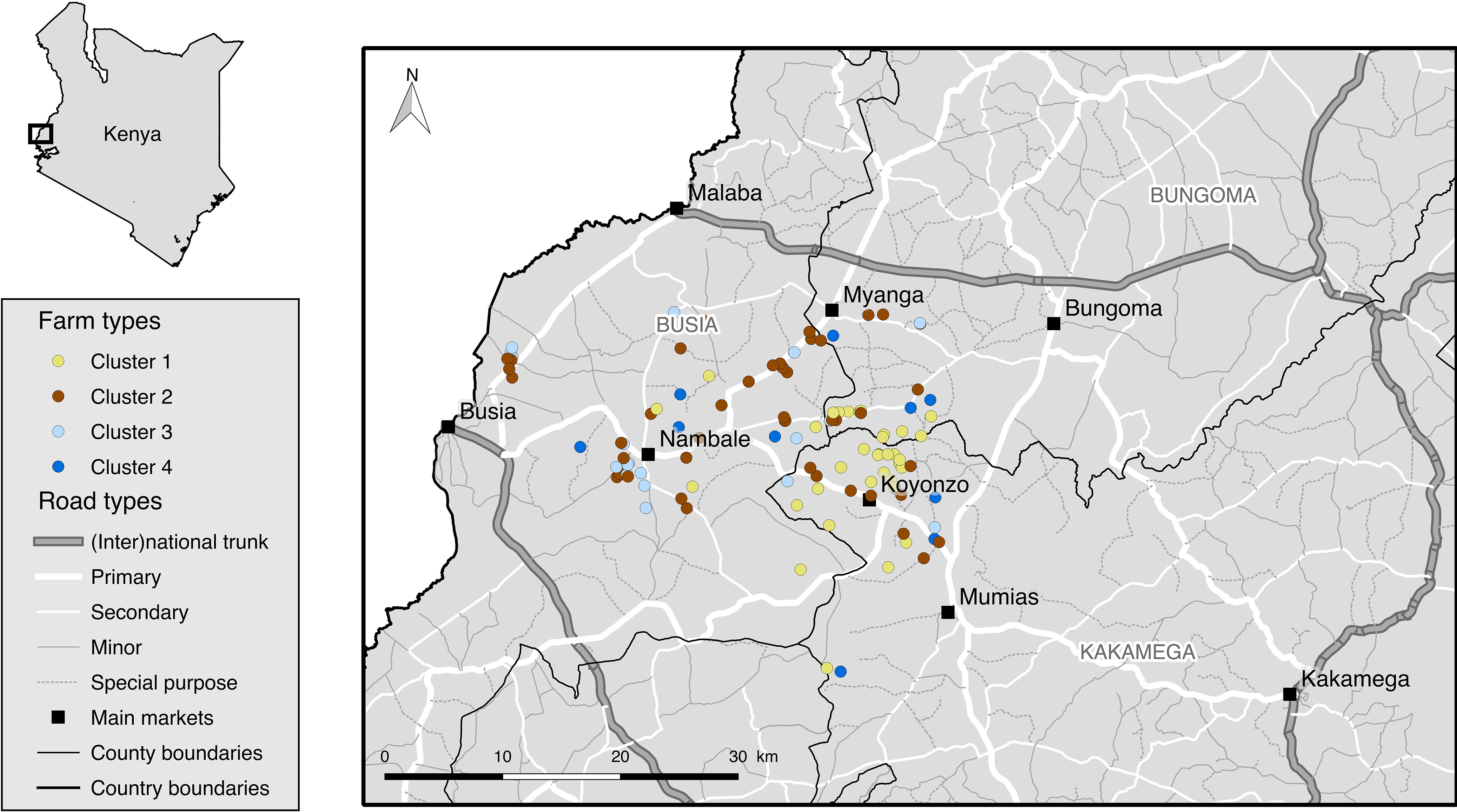
Spatial distribution of the chicken farm clusters, road network and main markets, within the study area of Western Kenya (Busia, Bungoma and Kakamega counties).

**Figure 2:**
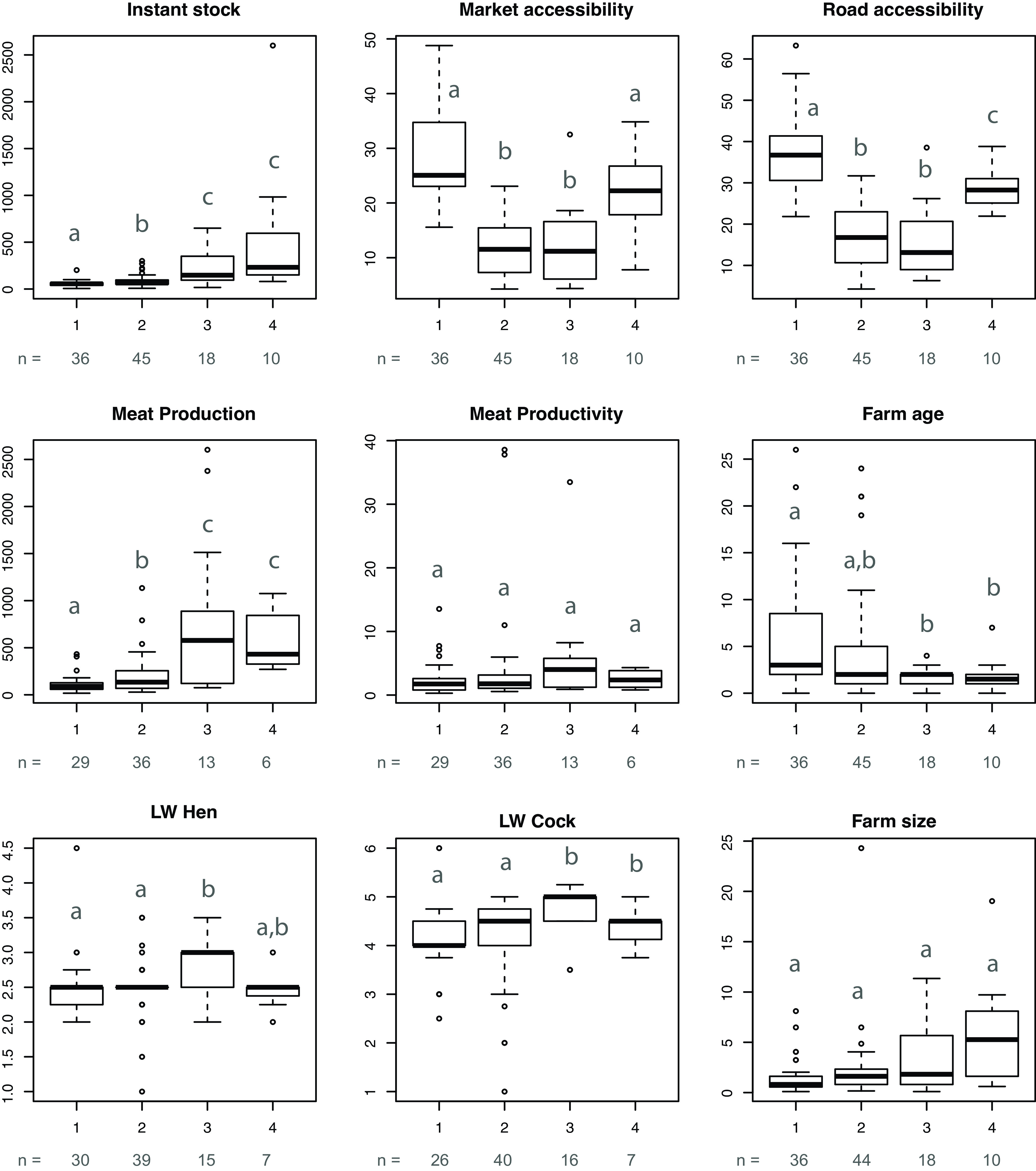
Box plots of quantitative variable by cluster. Instant stock (number of chickens at interview time), market accessibility (min), road accessibility (min), meat production (total kg of meat/farm/year), meat productivity (kg/chicken place/year), farm age (time since commercial activity started in years), live weight (LW) of cock and hen (kg) and farm size (ha). The letters denote significantly different means at the *P* = 0.05 level (Kruskal–Wallis rank sum test) and *n* is the total number of farms by cluster.

The spatial distribution of the clusters is represented on Figure 1. Clusters were characterized using the entire data set (Figure 2, Supplementary Material Table S2).

### Cluster 1: small-scale semi-intensive farms, isolated (36 farms)

The first group contains the smallest farms in our survey, in area and in flock size (56 chickens and 1.6 ha) (Supplementary Material Figure S3a, b and arrow 1, Figure 2). These farms were overall the oldest farms (average of 6 years), but farmers belonged to a broad age range. The farms in cluster 1 had the lowest meat and egg production and productivities (117 kg of meat/farm/year, 2.6 kg/chicken place/year, 4719 eggs/year and 177 eggs/hen place/year) (Figure 2, Supplementary Material Figure S4). Farms in cluster 1 were the most distant ones from the main roads and the markets (37 and 28 min) (Supplementary Material Figure S3a, b and arrow 2). Flocks were home produced and of local breeds (Supplementary Material Figure S3a, b and arrow 1). Nearly half of those farmers used their own crops and found their location advantageous in that they could grow feed (Supplementary Material Table S2). Home-produced feed crops in the area included maize, millet, soybeans and sorghum. Over half of their production was sold at the farm gate, with markets and restaurants being other important outlets. The main challenges these farmers faced were disease control, theft, predators and lack of funds.

### Cluster 2: medium-scale semi-intensive farms, accessible (45 farms)

Farms in cluster 2 were largely similar to those in cluster 1, although they had larger flocks (88 chickens, *P* < 0.05), slightly better meat production (*P* < 0.05) and productivity (209 kg of meat/farm/year and 4.3 kg/chicken place/year) and egg production (6431 eggs/year) but lower egg productivity (103 eggs/hen place/year) (Figure 2, Supplementary Material Figure S4). They also had slightly larger plots (2.3 ha) and were more recent (4 years) (Figure 2). More farms in cluster 2 than in cluster 1, exclusively used commercial feed (Supplementary Material Table S2). Farms in cluster 2, together with the farms in cluster 3, were the closest to the main roads and the markets (18 and 12 min) (*P* < 0.05, Supplementary Material Figure S3a, b and arrow 2, Table S2), and this is the group that mostly mentioned road accessibility as an advantage.

### Cluster 3: large-scale semi-intensive farms, accessible (18 farms)

Cluster 3 had larger flocks (*P* < 0.05) and more land (224 chickens and 3.2 ha) compared to clusters 1 and 2 (Supplementary Material Figure S3a, b and arrow 1, Figure 2). Their commercial activity was more recently developed (2 years, *P* < 0.05). More farms in this cluster raised improved breeds (Supplementary Material Figure S3a, b and arrow 1, Table S2). Improved breeds in the area included mainly kuroiler, kenbro and rainbow rooster. The production of farms in cluster 3 was larger (*P* < 0.05) and they had the highest production and productivity (782 kg of meat/farm/year, 5.9 kg/chicken place/year, 12 106 eggs/farm/year and 205 eggs/hen place/year) (Supplementary Material Table S2, Figure S4). They had accessible locations (16 and 12 min away from roads and markets) (Supplementary Material Figure S3a, b and arrow 2, Figure 2). The use of home-grown feed was less important, and the use of commercial feed was larger than in the small- and medium-scale farms (Supplementary Material Table S2). Unlike farmers in clusters 1, 2 and 4, who mostly produced their own chicks, most farmers in cluster 3 purchased chicks (Supplementary Material Table S2, Figure S3e and f). The prices of live chickens, hens and cocks were higher than in clusters 1 and 2 (*P* < 0.05, Supplementary Material Figures S5 and S6). Farm gate sales and sales to traders were also very important in this cluster (Supplementary Material Table S2). However, sales to other farmers and to a lesser extent sales to restaurants were more important than in small- and medium-scale farms. The main constraints faced by the farms from cluster 3 were feed cost and diseases. However, farmers reported more issues related to markets than farmers in clusters 1 and 2, and they viewed market availability as a major location advantage. Moreover, the farmers cited competition from cheap eggs from Uganda as a constraint.

### Cluster 4: large-scale semi-intensive, isolated (10 farms)

Farms in cluster 4 had the largest flocks and the most land (549 chickens and 6.1 ha) (Supplementary Material Figure S3a, b and arrow 1, Figure 2). Farms in cluster 4 were similar to cluster 3 but obtained their chicks from natural incubation (Supplementary Material Table S2, Figure S3c and d) and reported chick management (high mortality rate) and electricity supply as important challenges (Supplementary Material Table S2). They purchased all feed and prepared home-mixed feed, complemented or otherwise by commercial feed. Raw products in the area were bought separately from the market or from other farms and included cereals such as finger millet, maize, maize bran, rice, rice bran, sorghum and wheat but also supplements such as soybeans, beans, sunflower cake, cotton seed cake, dried omena fish (*Rastrineobola argentea*), fish meal, shell, mill by-products, Sukuma wiki (a cultivar of *Brassica oleracea*), shrimps or bones. They had slightly lower meat production and productivity (563 kg of meat/farm/year and 2.5 kg/bird/year) and egg productivity (146 eggs/hen place/year) but higher egg production (21 632 eggs/farm/year) (Figure 2, Supplementary Material Figure S4). They sold their chicken at a lower prices than farms in the other large-scale cluster (*P* < 0.05, Supplementary Material Figures S5 and S6). They also produced more diverse output products than cluster 3, selling chickens with eggs, chicks or both (Supplementary Material Table S2). These farms had poor accessibility to main roads and markets (29 and 23 min). As in cluster 3, farmers faced constraints like marketing. They cited low sale prices, insufficient demand, poor market access and poor road access as disadvantages of their location.

**Figure 3:**
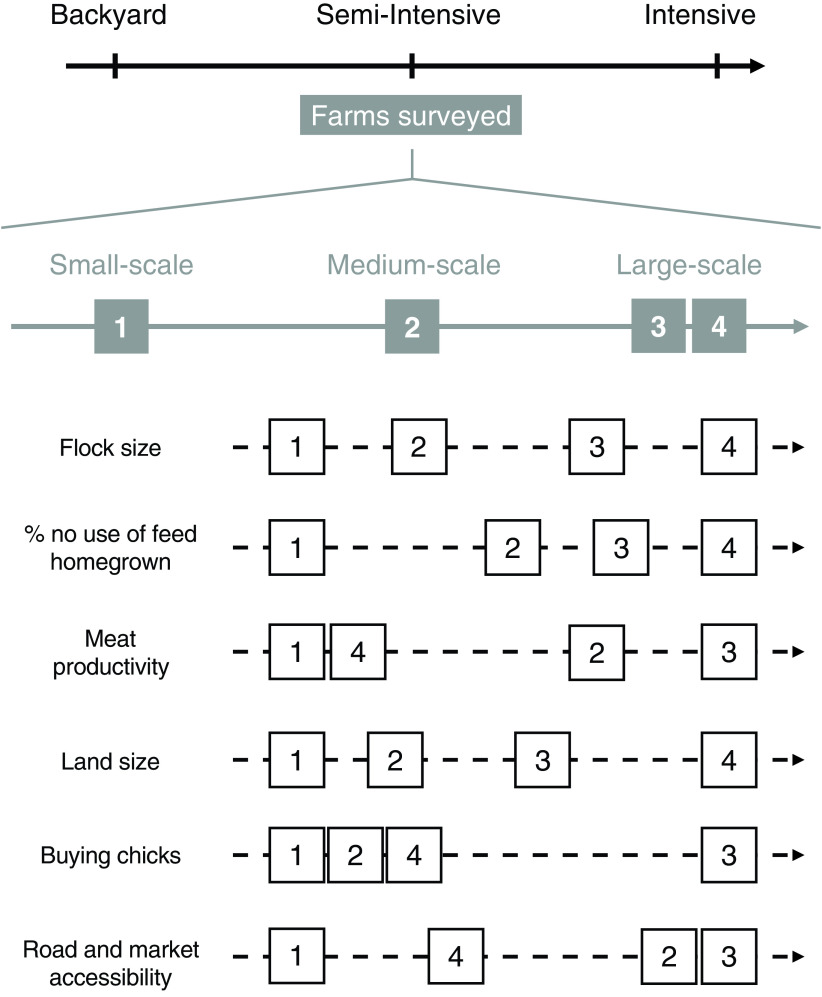
Chicken farm profiles along the gradient of intensification, from backyard to intensive systems, with a summary of the main characteristics of each farm profile along the intensification gradient. Numbers in square boxes refer to the four farm clusters.

The main cluster characteristics are graphically summarized in Figure 3. A gradient of intensification is expressed from clusters 1 to 4, considering flock size, the percentage of feed home-grown and land size. This trend is contrasted while considering meat productivity, purchase of chicks and road accessibility (Figure 3), with the lower values in farms of cluster 4.

Overall, no farm was solely dedicated to chicken production; all reported multiple sources of income. Among the chicken farmers sampled, 88% were also involved in crop farming, 68% in other livestock farming (cattle, goats, sheep, pigs, ducks or guinea fowl) and 45% in professional off-farm activity (e.g. teacher, police officer, employee). No farm had a marketing contract with a meat processor or an egg retailer. Retail and wholesale prices of chickens and eggs were similar, but they fluctuated as indicated by the minimum and maximum prices (i.e. hen sale prices in wholesale averaged 451 and 627 Kenyan shillings (Ksh)) (Supplementary Material Figure S7). Hatching eggs were more expensive than eggs for consumption (21 and 11 Ksh) (Supplementary Material Figure S8).

## Discussion

Poultry farming systems are typically organized along a gradient of intensification ranging from backyard farming to the most intensified and industrialized modes of production. Our survey aimed at characterizing small-scale commercial poultry production systems that are at very early stages of intensification, *that is*, when production shifts from subsistence to commercial systems, in a context of rising demand in rural areas. It sheds light on the diversity of management practices and the range of constraints faced by the farmers.

The farmers surveyed were unspecialized, raising dual-purpose breeds, without targeting any specific marketing outlets or feed sources, in contrast with more intensive systems (Oosting *et al.*, 2014; Binswanger-Mkhize and Savastano, 2017). Their main challenges were the cost of feed and the burden of disease, and poultry farming provided a rapidly accessible source of cash. These are typically characteristics of backyard production systems described in Kenya (Kingori *et al.*, 2010; Okeno *et al.*, 2012; Ochieng *et al.*, 2013; Mwobobia *et al.*, 2016) and other LMICs (FAO, 2014; Alders *et al.*, 2018). However, they generally kept larger flocks than backyard farms (Ochieng *et al.*, 2011), their production was mostly for sale and generated an income, they provided feed and their overall productivity (2 to 5 kg chicken meat/year/head place) was similar to semi-intensive farms (Alders *et al.*, 2018; ‘FAOSTAT’, 2019).

Farming in our sample was largely an individual endeavour without formal marketing link with large companies. We did not find any contract farmers such as encountered in large broiler companies from Nairobi (Carron *et al.*, 2017) or in other transition economies such as Thailand where large companies support small-scale farmers with inputs and market access (Alders and Pym, 2009). Their status as independent farmers may expose them to economic instabilities from the lack of financial capital and disease threats and make access to veterinary health services more difficult (Alders and Pym, 2009). However, they can balance those instabilities with other sources of income in crops and other livestock. In addition, their relatively low volumes of production and informal ways of marketing, which were already described in Kenya (Carron *et al.*, 2017; Onono *et al.*, 2018), allow them independence from middlemen and control over their own sales.

Notwithstanding these general characteristics, we found a high diversity of practices. We identified four clusters that differed in terms of scale of operation and accessibility and in many other ways. The bias due to the change in sampling method may have influenced the relative proportion of farms in the different clusters but not their characteristics and is thus unlikely to have influenced our farm typology.

The first two clusters, smallest in flock size (clusters 1 and 2), shared many characteristics and were both largely dependent on home-produced inputs. They differed mostly by their accessibility to markets. Their relatively low level of production may translate into an instable income. This instability may be exacerbated by inadequate management practices and low investment in poultry housing, which does not protect the chickens from theft or predation. These farms, the oldest in our sample, may have simply scaled backyard production up, with little adaptation. Their main challenge for future development may be the limited ability to invest further. The better market access of the medium-scale farms (cluster 2) may constitute a competitive advantage that could potentially allow them to continue developing their activity.

The two larger scale farms clusters (3 and 4) presented more typical characteristics of semi-intensive production systems (FAO, 2014; Alders *et al.*, 2018) but in diverse combinations (Figure 3). Their higher and regular production volumes allow them to establish business relationships with regular customers such as restaurants as well as the sale of chicks or fertilized eggs to other farmers. These larger farms also more frequently reported marketing issues, which are typical of small-scale intensive systems (Wong *et al.* 2017) and which may constitute a challenge to their future development. There was a noticeable difference between the two types of larger scale farms. Cluster 3, the large-scale farms with good accessibility that purchased chicks, presented the most classical characteristics of the intensified poultry production systems. They based their production on purchased inputs and reached the highest productivities and chicken selling prices. Their hens and cocks reached high live weights, thanks to having better genetic potential from the purchased chicks and/or using better quality feed. Their good locations in relation to markets reduced their dependence on middlemen, allowing them higher profit margins (Carron *et al.*, 2017). This suggests that they may be the most suitable for further development. The large-scale farms of cluster 4 were more isolated and home produced their chicks. These showed potential for development but faced greater constraints. Their remote location forced them to produce with a low dependence on external inputs. They hatched their own chicks with the specific challenges of chick management, such as, for example, the reliability of electricity supply to incubate the eggs. However, these farmers had larger plots that they could use to produce their own feed and that provided alternative and more diversified sources of income. For these more remote farmers, diversification of income sources with a larger choice of products can be an interesting livelihood strategy (Scoones, 1998), which has been shown to increase household welfare (Binswanger-Mkhize and Savastano, 2017). However, despite high stocking rates, their annual total meat production and productivity, and chicken selling prices were lower than in cluster 3, which may be due to a lower conversion rate of their home-mixed feed. Similarly to our results, households in market-constrained situation showed a need for larger land to achieve sufficient food availability (Frelat *et al.*, 2016). However, this may not be sustainable as farm size tends to decline in sub-Saharan African (Loison, 2015). Overall, their lower productivity and lower market access may compromise their sustainability in the long run, but this may be balanced by their higher resilience linked to more diverse types of production.

Generally, backyard poultry farming dominates in Western Kenya (Kingori *et al.*, 2010; Ochieng *et al.*, 2011; Okeno *et al.*, 2012), but we observed many farms with characteristics of semi-intensive farming. They presented a complex spectrum of intensification. Generally, the larger the flock size, the higher the productivity, sale prices and use of improved breeds and commercial feed. However, we observed nuances along this intensification gradient. Cluster 4 had lower productivity and market accessibility, inconsistently with the characteristics of intensive farms (Aklilu *et al.*, 2008; Binswanger-Mkhize and Savastano, 2017). It also highlighted a nuanced response of farm intensification to market accessibility. Farmers adapted their management to the local conditions, and this resulted in a great diversity of farms presented in Figure 3. This statement should, however, be taken carefully, as the cross-sectional nature of the data prevents from analysing the intensification process itself. While our cross-sectional data does not allow firm interpretation of trajectories, the age of farms suggests that some may evolve gradually from backyard flocks (clusters 1 and 2) and struggle to invest solidly in intensive operations, while others may have started, recently, with semi-intensive features (clusters 3 and 4).

Our results outline a number of questions on the development of intensive chicken production in rural areas. The largest farms (clusters 3 and 4) had been established recently, which raises many questions on the conditions for establishment and what the future may hold for them. These farms may be particularly unstable economically, with many farmers starting or stopping their activity. This high turnover may explain their recent status. Alternatively, the intensification of poultry production may simply have occurred recently in this area. This may have resulted from recent increases in local demand and the opening of new business opportunities. The increase in demand may continue in the future, allowing these farmers to keep intensifying. Increases in local demand are particularly likely in the current policy of decentralization. Further urbanization of county capitals and local centres, such as the towns of Kakamega, Bungoma and Busia, is expected to continue in Kenya. Local and sustainable intensification could be further supported by targeted training programs that would take into account the existing heterogeneity of semi-intensive poultry production systems. To further help local smallholders to increase their productivity, innovations matching their local production conditions should be promoted.

Competition in the poultry sector, even in those remote areas, may become stronger in the future. Potential future improvements to infrastructure would reduce transport costs and allow large industrial poultry production farms based around the main urban centres to transport their products to Western Kenya and ultimately outcompete local producers. This has been observed in pig production in Thailand, where industrial production located around the main urban centres (e.g. Bangkok) outcompetes backyard or small-scale producers who gradually disappear (Thanapongtharm *et al.*, 2016). Free trade between East African countries could also flood this area with poultry produced in other countries. This is apparently already the case with egg production in our study area, with several farmers complaining about competition from cheap eggs imported from Uganda.

The future of local large-scale production may also be compromised by the difficulty in shifting from semi-intensive to intensive farming targeted at urban consumers. The shift from backyard to semi-intensive production generally results from individual entrepreneurship and personal skills. However, the transition from semi-intensive to intensive farming is neither gradual nor frequent (Oosting *et al.*, 2014). It requires structural change in terms of investments in infrastructures, access to inputs and trade of outputs such as to become highly market-oriented and increasingly specialized. Furthermore, profit margins have to be shared with many players along the value chain. The main bottleneck is investment capacity. As a result, smallholders intensifying in the context of a rise in demand for animal-source food are usually not the poorest but the better-off farmers (Udo *et al*., 2011). The process of intensification is likely not a linear one affecting all farms but rather probably also involving replacement when economically stronger actors seize new opportunities. Intensification of poultry production in Kenya, as in many other LMICs, may take the form of intensive systems being located in the surroundings of old or new urban centres, with backyard and semi-intensive systems persisting in rural areas until they are outcompeted.

The poultry sector in Western Kenya is still at the early stages of intensification and diverse farmers coexist and share the informal chicken market. Understanding how the local conditions influence production types is essential to support the livelihood of small-scale farmers and to help them develop a sustainable and profitable agricultural production. Today, the spatial factors influencing the intensifying production systems of LMICs are poorly known, and longitudinal data are rarely available. This hinders the understanding of the intensification process as part of the economic development in rural areas (Loison, 2015). This is particularly important as the intensification process in sub-Saharan African countries differs from the structural transformation path and economic development followed in the past by high-income countries (Loison, 2015).

This study highlights the importance of local conditions on the development of production management types. The farms surveyed generated income through chicken production and displayed many features of semi-intensive systems. Our results open interesting questions about the evolution of small-scale commercial farms as a result of income growth, infrastructure improvement and competition from imports of urban production. It also questioned the linearity of the intensification process, as it seemed to give rise to diverse farm types rather than a gradual evolution.

## Software and data repository resources

Data are publicly available in The Research Data Catalogue of the University of Liverpool at the following link: https://doi.org/10.17638/datacat.liverpool.ac.uk/1066.

## Supplementary material

For supplementary material accompanying this paper visit https://doi.org/10.1017/S175173112000110X. click here to view supplementary material
