# Supplementary material for: Early intensification of backyard poultry systems in the tropics: a case study
Source: Animal. 2020 Jun 24;14(11):2387–96. doi: 10.1017/S175173112000110X (PMC7538343; doi:10.1017/S175173112000110X)
Supplement: Supplementary file 1 [file S175173112000110Xsup001.docx]

**Supplementary materials to**

**Early intensification of backyard poultry systems in the tropics – a case study**

*animal* journal

Celia Chaiban, Timothy P. Robinson, Eric M. Fèvre, Joseph Ogola, James Akoko, Marius Gilbert and Sophie O. Vanwambeke

**Method**


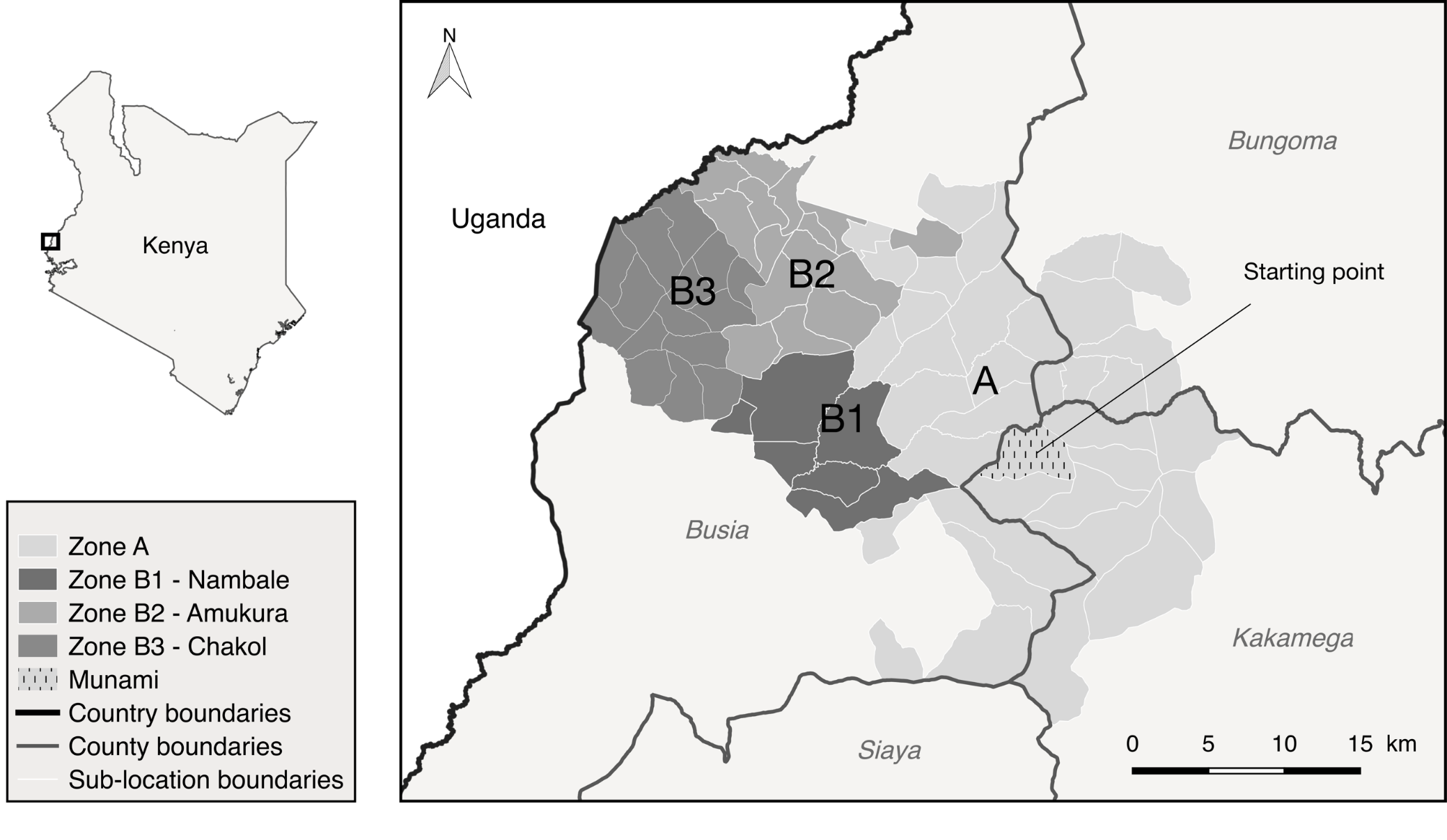


**Figure S 1.** Study area across Busia, Bungoma and Kakamega counties, in western Kenya and sampling methods of chicken farm data collection. The census of all commercial chicken farms was conducted following a snowball sampling approach, spiralling out of the starting sublocation (Munami sublocation) and going from one assistant chief to another to reach farmers. Each farm was visited with either the assistant chief (sub-location administrative officer) or a village elder (Zone A – first sampling method). In five sub-locations from Nambale wards (the ones which were not visited yet) (Zone B1), in 15 sub-locations of Amukura ward (Zone B2, the ones which were not visited yet) and all sub-locations of Chakol ward (Zone B3), ward livestock production officers were consulted (Zones B – second sampling method).

*Statistical analysis: Farm typology*

We used eight variables to define farm profiles: (i) instant stock; (ii) annual number of birds slaughtered; (iii) accessibility to main roads (minutes) (iv) accessibility to main markets (minutes) (v) farms with improved breeds and (vi) farms with local breeds (indigenous chickens); and chick source, (vii) farmer home-producing chicks or (viii) farmer purchasing chicks (Table 1 in the manuscript). We performed the Principal Component Analysis (PCA) using the *PCAmixdata* package in R software. We selected Principal Components (PC) according to the scree plot of Eigen values and the total variability explained (Figure S2 a). The first three PC had Eigen values over one and represented 72% of the variability.

Ward’s Minimum Variance Clustering was implemented using the *hclust* function from the R package *stats* with *method=“ward.D2”* ^[[1]](#footnote-1)^ and K-means partitioning using *kmeans* function from *stats*. As an input, Ward clustering requires a distance matrix while K-means works directly with the variables. The input in our Ward clustering was an Euclidean distance matrix calculated on the three PCs, and in our K-means, was the three first PCs. To choose the number of clusters, we offset the within-cluster similarity against the number of clusters (Figure S2 c, d). The within cluster similarity was represented by the dendrogram height for Ward method and the within cluster sum of squares for K-means method.

*Questionnaire data processing for cluster characterisation*

Open questions (such as feed types, advantages and disadvantages of farm location or constraints) were classified in different types as indicated in Table 1 in the manuscript according to farmers’ answers. By cluster, we considered the mean and median of each quantitative variable and the percentage of farms by category for each qualitative variable.

*Meat production variable*

The annual meat production was defined as $Meat production=St.Ot.Cw$ (kg of meat/farm/year), where *St* is the instant stock, *Ot* the offtake rate and *Cw* the carcass weight. As the offtake rate was not available, we used the annual number of birds slaughtered (Bs) following $Bs = St.Ot$.Carcass weight (*Cw*) was $Cw=Lw. Dp$, where *Lw* is the estimated live weight and *Dp* the dressing percentage. The dressing percentage was set to 72% according to Kingori *et al.* (2010)^[[2]](#footnote-2)^.

*Accessibility variables*

**The accessibility to main roads**, was an accessibility to international and national paved trunk roads (road classes A and B). It was computed using a travel time (minutes) to the closest intersection between those A and B road classes and the rest of the road network (see Figure 2 in the manuscript for a geographical representation of the road network within the study area). The travel time was computed based on the speed weighted road network^[[3]](#footnote-3)^. First, we computed a cost surface, in which weights were assigned to each road class. Weights were based on the different road speeds (Table S 1): $Time \left( s \right) to travel one meter= \frac{360}{speed(\frac{km}{h})}$. Second, we used a cost allocation to compute a space allocation to each intersection (between A and B road classes and the rest of the road network). The accessibility computation was implemented with ArcMap software and using the geographic projection World Geodetic System (WGS) 84 – Universal Transverse Mercator (UTM) 37 North zone.

**The accessibility to main markets** was computed in the same way; a travel time (minutes) to the closest markets among Koyonzo, Mumias, Nambale, Busia, Malaba, Myanga, Bungoma, Kakamega markets (see Figure 2 in the manuscript for a geographical representation). The main markets corresponded to the markets most frequented by the farmer from this study.

**Table S 1.** Road speeds assigned to each road type in the study area. These road speeds were used to compute travel time of each chicken farm to main roads or main markets.

| Road class | Road type | Road speed (km/h) | Weight assigned |
| --- | --- | --- | --- |
| A | International trunk Roads | 110 | 3 |
| B | National trunk Roads | 110 | 3 |
| C | Primary Roads | 70 | 5 |
| D | Secondary Roads | 50 | 7 |
| E | Minor Roads | 30 | 12 |
| G | Minor Government Roads | 30 | 12 |
| L | Minor Settlement Roads | 30 | 12 |
| R | Special Purpose Roads – Rural Access Roads | 15 | 24 |
| S | Special Purpose Roads – Sugar Roads | 15 | 24 |
| T | Special Purpose Roads – Tea Roads | 15 | 24 |
| U | Unclassified Roads | 10 | 36 |
| / | No data | 5 | 72 |

**Results**

**Figure S 2.** Results of clustering analysis performed on chicken farm data. (a) Eigen value of the different Principal Components (PC), the percentages represent the variability explained by each PC. (b) Biplot on the two first PC. The four colors of the points represent the different clusters with K-means method, the color of the circle indicates the cluster which the farm was assigned to with ward clustering. The farms spread more widely along the PCA dimensions with the K-means method. Within cluster similarity against the number of clusters (c) K-means clustering method and (d) Ward clustering method.


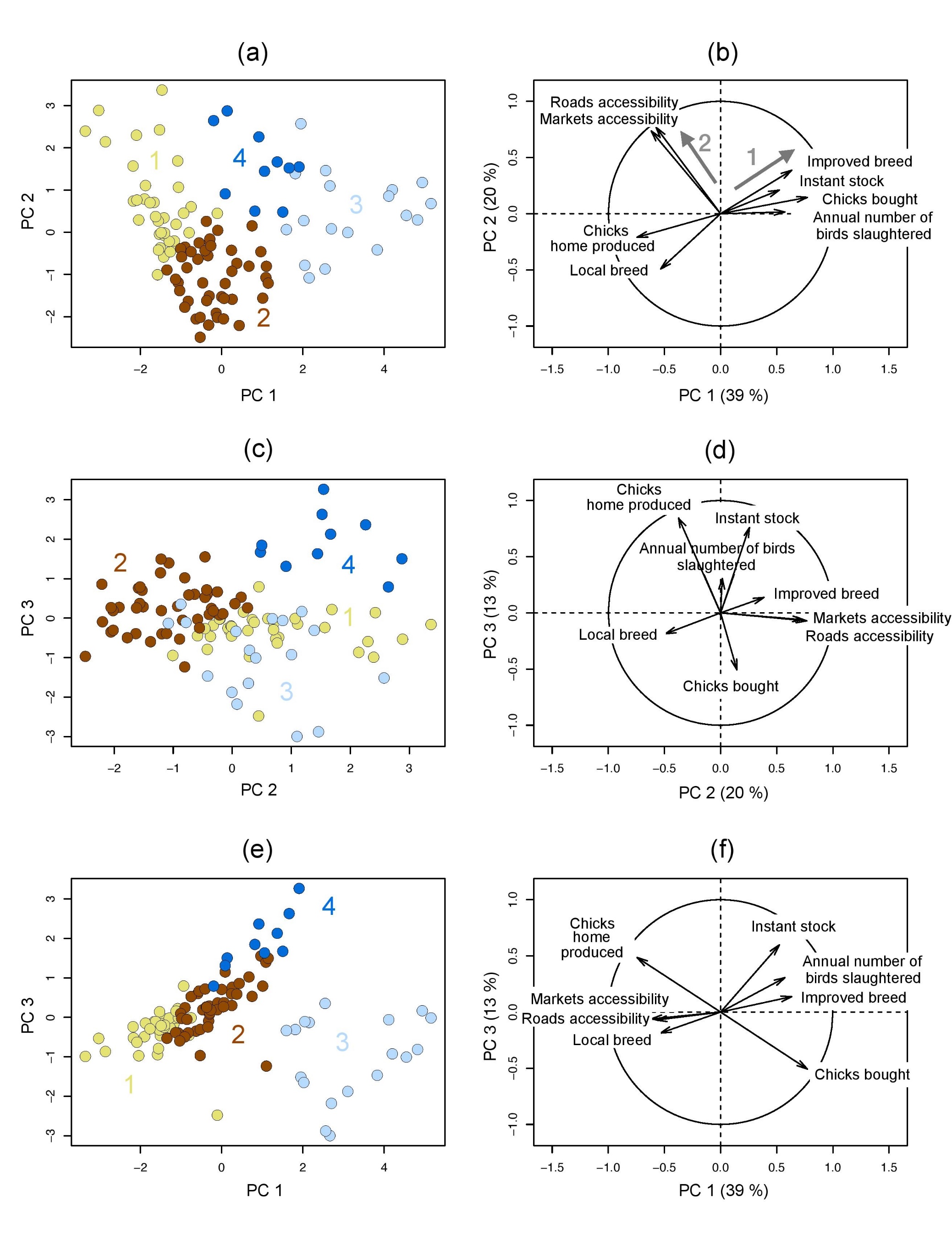


Figure **S 3.** Results of the Principal Component Analysis (PCA) and the K-mean clustering method, performed on the chicken farm data. Biplot of the observations (chicken farms) by cluster on the first principal components (PC) (a), on PC2 and PC3, (c) on PC1 and PC3 (e). A colour was assigned to each cluster (as indicated by the cluster number). Projection of the variables used in the PCA, on the two first PC (b), on PC2 and PC3 (d) and on PC1 and PC3 (f).

Table S 2. Summary statistics for the entire dataset and the four clusters of chicken farms. The star indicates the variable for which multiple answers were possible. In these cases, a farm can therefore be accounted several times and the sum of percentages can be above one hundred. The variables are defined as followed. Breed type, farms having local or improved breeds or both. Chick source, farms having chick home-produced (Produced), bought or both. Products sold, farms selling chickens, eggs or chicks. Feed type includes commercial feed (CF), home-produced feed (HP), raw products (RP). Types of output destination include farm gate, trader, market, farmer, restaurant, other (includes school, shop, slaughterhouse, butcher)). Advantages of farm location include market access, market available, feed access, road access, using their own crop as feed (own crops). Disadvantages of farm location include insufficient demand, low market access, low road access, low prices of sold products (low prices), disease and theft of chickens. Constraints cited by farmers include feed cost, disease, lack of knowledge on chick management (chick management), lack of knowledge on poultry keeping management (knowledge), electricity, vaccine and drug cost (drug cost), theft, predation (predators), low prices of Ugandan eggs (cheap egg < Uganda), instability of market (marketing issues), and lack of fund.

|  | **All data** | | **Cluster 1** | | **Cluster 2** | | **Cluster 3** | | **Cluster 4** | |
| --- | --- | --- | --- | --- | --- | --- | --- | --- | --- | --- |
|  | n | N (prop) | n | N (prop) | n | N (prop) | n | N (prop) | n | N (prop) |
| **Breed types**  Local  Local & Improved  Improved | 109 | 58 (0.53)  33 (0.30)  18 (0.17) | 36 | 28 (0.78)  7 (0.19)  1 (0.03) | 45 | 29 (0.64)  15 (0.33)  1 (0.02) | 18 | 1 (0.06)  9 (0.5)  8 (0.44) | 10 | 0 (0)  2 (0.20)  8 (0.80) |
| **Chick source**  Produced  Produced and bought  Bought | 109 | 88 (0.81)  7 (0.06)  14 (0.13) | 36 | 34 (0.94)  1 (0.03)  1 (0.03) | 45 | 44 (0.98)  1 (0.02)  0 (0) | 18 | 0 (0)  5 (0.28)  13 (0.72) | 10 | 10 (1.00)  0 (0)  0 (0) |
| **Products sold** | 109 |  | 36 |  | 45 |  | 18 |  | 10 |  |
| Chicken  Chicken & Egg  Chicken & Egg & Chick  Chicken & Chick  Chick |  | 54 (0.5)  32 (0.29)  11 (0.10)  10 (0.09)  2 (0.02) |  | 20 (0.56)  10 (0.28)  2 (0.06)  2 (0.06)  2 (0.06) |  | 26 (0.58)  11 (0.24)  4 (0.09)  4 (0.09)  0 (0) |  | 7 (0.39)  8 (0.44)  1 (0.06)  2 (0.11)  0 (0) |  | 1 (0.10)  3 (0.30)  4 (0.40)  2 (0.20)  0 (0) |
| **Feed type**  CF  CF & HP  CF & RP  CF & RP & HP  HP  RP  RP & HP | 109 | 28 (0.26)  28 (0.26)  21 (0.19)  16 (0.15)  1 (0.01)  9 (0.08)  6 (0.06) | 36 | 5 (0.14)  16 (0.44)  5 (0.14)  5 (0.14)  1 (0.03)  3 (0.08)  1 (0.03) | 45 | 12 (0.27)  9 (0.20)  8 (0.18)  7 (0.16)  0 (0)  5 (0.11)  4 (0.09) | 18 | 8 (0.44)  3 (0.17)  4 (0.22)  2 (0.11)  0 (0)  0 (0)  1 (0.06) | 10 | 3 (0.30)  0 (0)  4 (0.40)  2 (0.20)  0 (0)  1 (0.10)  0 (0) |
| **Types of output destination*** | 109 |  | 36 |  | 45 |  | 18 |  | 10 |  |
| Farm gate  Trader  Market  Farmer  Restaurant  Other |  | 64 (0.59)  44 (0.4)  30 (0.28)  31 (0.28)  28 (0.26)  19 (0.17) |  | 20 (0.55)  17 (0.47)  12 (0.33)  6 (0.17)  7 (0.19)  6 (0.17) |  | 27 (0.60)  16 (0.36)  12 (0.27)  9 (0.20)  11 (0.24)  6 (0.13) |  | 13 (0.72)  8 (0.44)  3 (0.17)  9 (0.50)  5 (0.28)  6 (0.33) |  | 4 (0.40)  3 (0.30)  3 (0.30)  7 (0.70)  5 (0.50)  1 (0.10) |
| **Location advantages***  Market access  Market available  Feed access  Road access  Own crops | 104 | 27 (0.26)  52 (0.50)  8 (0.08)  12 (0.12)  15 (0.14) | 35 | 8 (0.23)  17 (0.49)  3 (0.09)  3 (0.09)  10 (0.29) | 42 | 12 (0.29)  18 (0.43)  3 (0.07)  8 (0.19)  5 (0.12) | 18 | 7 (0.39)  10 (0.56)  2 (0.11)  1 (0.06)  0 (0) | 9 | 0 (0)  7 (0.78)  0 (0)  0 (0)  0 (0) |
| **Location disadvantages***  Insufficient demand  Market access  Road access  Low prices  Disease  Theft | 103 | 6 (0.06)  8 (0.08)  2 (0.02)  18 (0.17)  9 (0.09)  20 (0.19) | 36 | 1 (0.03)  2 (0.06)  1 (0.03)  6 (0.17)  5 (0.14)  7 (0.19) | 42 | 3 (0.07)  4 (0.10)  0 (0)  7 (0.17)  2 (0.05)  12 (0.29) | 16 | 1 (0.06)  1 (0.06)  0 (0)  3 (0.19)  2 (0.13)  1 (0.06) | 9 | 1 (0.11)  1 (0.11)  1 (0.11)  2 (0.22)  0 (0)  0 (0) |
| **Constraints*** | 109 |  | 36 |  | 45 |  | 18 |  | 10 |  |
| Feed cost  Disease  Chick management  Knowledge  Electricity  Drug cost  Theft  Predators  Cheap egg < Uganda  Marketing issues  Lack of fund |  | 43 (0.39)  73 (0.67)  16 (0.15)  5 (0.05)  5 (0.05)  5 (0.05)  31 (0.28)  20 (0.18)  3 (0.03)  14 (0.13)  22 (0.20) |  | 17 (0.47)  27 (0.75)  6 (0.17)  3 (0.08)  0 (0)  1 (0.03)  11 (0.31)  8 (0.22)  1 (0.03)  3 (0.08)  8 (0.22) |  | 15 (0.33)  30 (0.67)  7 (0.16)  0 (0)  2 (0.04)  2 (0.04)  16 (0.36)  8 (0.18)  0 (0)  6 (0.13)  13 (0.29) |  | 6 (0.33)  11 (0.61)  0 (0)  1 (0.06)  0 (0)  1 (0.06)  2 (0.11)  3 (0.17)  2 (0.11)  3 (0.17)  1 (0.06) |  | 5 (0.50)  5 (0.50)  3 (0.30)  1 (0.10)  3 (0.30)  1 (0.10)  2 (0.20)  1 (0.10)  0 (0)  2 (0.20)  0 (0) |

**Figure S 4.** Boxplots of the annual number of birds slaughtered (number of sold chickens/year), egg production (number of eggs/year), and the egg productivity (number of eggs/hen place/year) by cluster. The letters denote significantly different means at the p = 0.05 level (Kruskal-Wallis rank sum test), and *n* denotes the number of farms by cluster.

**Figure S 5.** Live hen prices received by farmers per cluster in Kenyan shillings (Ksh) (1 US$ = 101.33 Ksh). Prices are provided by sale type (wholesale (Whsl) and retail) and price type (usual, maximum and minimum price). The letters denote significantly different means at the p = 0.05 level (Kruskal-Wallis rank sum test), and *n* denotes the number of farms by cluster.

**Figure S 6.** Live cock prices received by farmers per cluster in Kenyan shillings (Ksh) (1 US$ = 101.33 Ksh). Prices are provided by sale type (wholesale (Whsl) and retail) and price type (usual, maximum and minimum price). The letters denote significantly different means at the p = 0.05 level (Kruskal-Wallis rank sum test), and *n* denotes the number of farms by cluster.

**Figure S 7.** Live chickens prices received by farmers in Kenyan shillings (Ksh) (1 US$ = 101.33 Ksh). Prices are provided for hen and cocks sold, by sale type (wholesale (Whsl) and retail) and price type (usual, maximum and minimum price), and *n* denotes the total number of farms.

**Figure S 8.** Chicken egg prices received by farmers in Kenyan shillings (Ksh) (1 US$ = 101.33 Ksh). Prices are provided for egg dedicated to consumption and hatching eggs dedicated to chicken production, by sale type (wholesale and retail), and price type (price at time of interview (usual), maximum and minimum price). *n* denotes the total number of farms.

1. Murtagh F and Legendre P 2014. Ward’s hierarchical agglomerative clustering method: which algorithms implement Ward’s criterion? Journal of Classification 31, 274–295. doi:10.1007/s00357-014-9161-z. [↑](#footnote-ref-1)
2. Kingori AM, Wachira AM and Tuitoek JK 2010. Indigenous chicken production in Kenya: A review. International Journal of Poultry Science 9, 309–316 [↑](#footnote-ref-2)
3. Road source: Kenya government Ministry of Roads and Public Works in collaboration with the World Bank [↑](#footnote-ref-3)
